# Veteran trees are a source of natural enemies

**DOI:** 10.1038/s41598-020-75723-0

**Published:** 2020-10-28

**Authors:** Ross Wetherbee, Tone Birkemoe, Anne Sverdrup-Thygeson

**Affiliations:** Faculty of Environmental Sciences and Natural Resource Management, Norwegian University of Life Sciences, P.O. Box 5003, 1432 Aas, Norway

**Keywords:** Community ecology, Conservation biology, Ecosystem services

## Abstract

Predation of invertebrate pest by natural enemies is a critical contribution of nature to people, because invertebrate pests cause a vast amount of economic damage and pesticides use has many long-term costs. Veteran trees are keystone structures and hotspots for biodiversity, and are a potential source of natural enemies. To explore this, we used a balanced experimental design where we measured predatory beetle diversity and attack marks on three colors of artificial caterpillars placed around 20 veteran oaks and 20 nearby young oaks, in Southern Norway. We predicted that around the veteran oaks there would be a greater diversity of predatory beetles and more invertebrate attacks on artificial caterpillars. Sampling for predatory beetles was conducted in summer 2017 and 2018, and invertebrate attacks were measured in 2018. We found support for the predictions: diversity of predatory beetles was higher around veteran trees and there were more arthropod attack marks on artificial caterpillars placed around veteran trees. Our results indicated that veteran trees are a source of natural enemies. Valuing and protecting veteran trees and their communities is an essential step towards a more sustainable system of management that has the possibility of promoting both the wellbeing of people and biodiversity.

## Introduction

Biodiversity loss and the associated environmental and social problems are considered some of the central challenges of our time^1^, in part, because all societies are dependent on the functioning of ecosystems for the support of human existence and wellbeing^2^. Nature’s contribution to people [NCP] has been suggested as a framework to help societies better understand and relate to the ecosystems on which they depend^3^. NCP can be defined as all the contributions, both positive and negative, of living nature (diversity of organisms, ecosystems, and their associated ecological and evolutionary processes) to people’s quality of life^1^.

Invertebrate pests are an example of an NCP with considerable negative impacts on society. Agricultural intensification has exacerbated this problem by simplifying the landscape and reducing biodiversity^4^, and the pervasive method of using pesticides to control invertebrate pests has many interrelated costs for local people, future generations and biological communities^5–8^. One viable alternative to the use of pesticides is to preserve or enhance predation by the natural enemies in a target area^9^. While pest control with natural enemies is an ancient method, with records of it being implemented as early as 900 AD^9^, it has recently gained new interest as an beneficial NCP^10,11^. Additionally, research has shown that landscape complexity is critical for this NCP, because higher levels of habitat heterogeneity have positive effects on the ability of multiple enemies to coexist due to the presence of additional non-pest prey and greater range of microhabitats^11,12^.

Veteran trees have played a prominent role in many cultures around the world and throughout the ages^13^. They are ‘keystone structures’ for biological communities^14,15^, and are an integral aspect in many traditional landscapes and sacred sites^13,16,17^. Veteran oaks are a hotspot for biodiversity in Northern Europe^18,19^ and enhance the structural complexity of the landscape^20^. Large trees in agroforestry systems have been found to enhance functional biodiversity and promote beneficial NCP including invertebrate pest control^21^. Veteran oaks provide food resources and shelter for a diverse set of species^19^, including arthropod predators^22,23^ and may be a source of a natural enemies of invertebrate pests.

However, high levels of biodiversity do not necessarily result in enhanced ecosystem functioning; ecosystem functioning is instead more closely related to a diversity in traits [functional diversity] rather than taxonomic diversity^24,25^. Therefore, functional diversity can be considered the link between biodiversity and ecosystem functioning. When specifically considering predation by natural enemies, mounting evidence suggests that predation increases when the natural enemies have complementary traits^10,11^. Natural enemies are complementary when they attack different pest species, have differences in their phenologies and diurnal/nocturnal activities, and have different hunting behaviors^11^. Although it has been established that veteran trees increase the structural complexity of the landscape^15^ and are a source of diverse predator^22^, the link between biodiversity and ecosystem functioning has received less attention and it is unknown to what extent the presences of these trees influences the beneficial NCP of predation by natural enemies.

Assessing differences in the predation by natural enemies is not straight forward^26^. Attacks by predators on invertebrate prey are usually cryptic and rarely leave any evidence of the event, and visual observations are seldom possible and complicated by presence of the observer^27^. Other methods such as examination of predator gut contents, or radioactive labelling of the prey have difficulties distinguishing between “real” predation and scavenging or secondary predation^28^. An alternative method of measuring predation intensity is with the use of artificial caterpillars. The technique involves fashioning prey from malleable, non-hardening material, deploying them in the field and then measuring depressions left on the models by predators^29,30^. The marks left on the artificial caterpillars from attempted predation event can be used to identify the predator^30^. Additionally, different colors of artificial caterpillars can be deployed to mimic different prey species^27,31^. The level of identification of predator attack marks has varied between studies, but identification at a coarse taxonomic level (bird, mammal, or invertebrates) has been shown to be the most prudent approach^29^. Invertebrate attacks on artificial caterpillars have been attributed to ants, ground beetles, predatory bugs, predatory wasps, parasitoid wasps, spiders^27^ and in some cases non-predatory insects^26,27,31^. Although the method of deploying artificial caterpillars to measure predation rates has limitations, it has been found to be suitable for measuring predation rates in comparative studies^27^.

In order to measure the contribution of veteran oaks to the beneficial NCP of predation by natural enemies, we employed a balanced experimental design. We matched twenty veteran oaks with twenty nearby young oaks, which were taken to represent the background levels, and measured the diversity of predatory beetles and the number of invertebrate attack marks on artificial caterpillars placed around the trees. We included trees from open landscapes and forests to span the variation that is observed in veteran oak distribution in Northern Europe, but we were primarily focused on the dichotomy between the veteran and the young tree. The study had two main predictions related to this dichotomy: first, there would be a greater diversity of beetle predators around veteran trees, and second, there would be more attacks on artificial caterpillars deployed around veteran trees. The first prediction was based on the fact that veteran oaks have high diversity of arthropods associated with them^19^. We defined diversity as including both taxonomic and functional diversity, and for our measure of functional diversity we chose to focus on traits that were identified as being complementary for natural enemies^10,22,32^. The next prediction was rooted in the first: a community of predators with diverse complementary traits (ie: predators with different hunting strategies, prey species and phenologies) will have high predation levels. If these predictions are supported, veteran trees should be considered as enhancing predation by providing a source of natural enemies.

## Results

**Figure 1:**
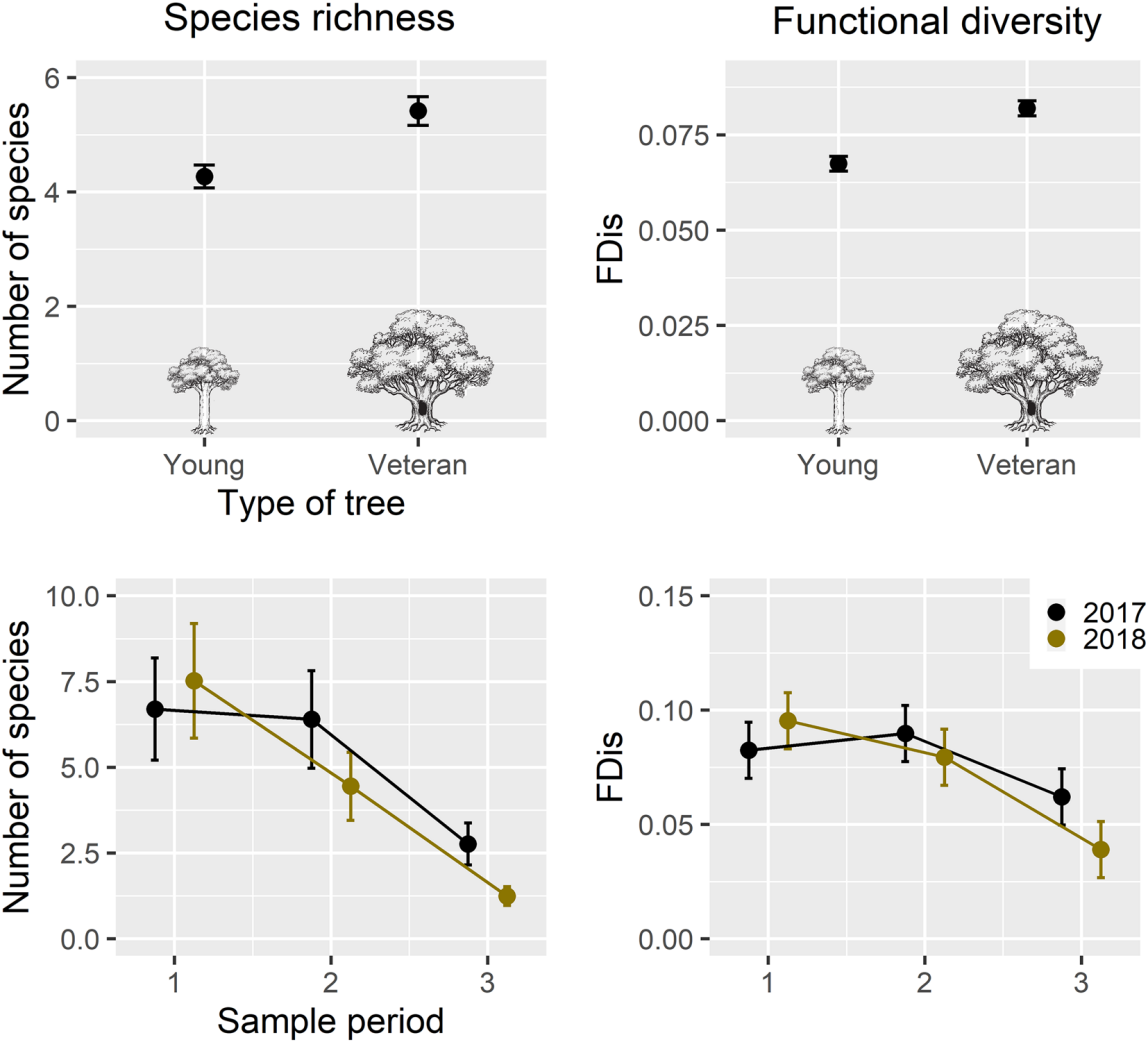
Estimates from the models that predicted species richness (left plot) and functional diversity (right plot) of predatory beetles (± SEM; n = 238 from 40 trees). Species richness was modeled with a generalized liner mixed effect model with Poisson error distribution, and functional diversity was calculated as functional dispersion (FDis) and modeled with a Liner mixed effect model with Gaussian error distribution. Both measures of diversity were higher around veteran trees (P = 0.01, 0.037, top), and independent of the type of tree there was a yearly and seasonal effect where diversity decreased through the season and was significantly lower at the end of 2018 (P = 0.001, 0.017, bottom). Window trap samples were collected once a month from May to August 2018, at the same time as the artificial caterpillars were collected. A summary of the models is presented the Table 1 (Ill. by Matthew Cooper).

Over the course of the two summers we captured a total of 465 beetle species of which 173 were predators. Both species richness and functional diversity of complementary traits were higher around veteran than young trees (Table 1, P = 0.01 and 0.037, respectively). Independent of the type of tree, there was a yearly and seasonal effect on both measures of diversity, which decreased through the season and was significantly lower at the end of the summer in 2018 (Table 1, Fig. 1).

**Table 1:** Summary of the optimal Linear (LMM) and Generalized linear (GLM) mixed effect models predicting species richness (GLM, Poisson error distribution) and functional diversity (LMM, Gaussian error distribution) of predatory beetles, and invertebrate attack rates (GLM, Negative binomial error distribution) on artificial caterpillars related to veteran and young oaks in Southern Norway.

Response variable and predictors		Estimate	Standard error	P value	Pseudo R2
**Species richness**					0.7
Intercept		1.781	0.087	**< 0.001**	
Type of tree	(Veteran)	0.235	0.095	**0.010**	
Sampling period	(Middle) (last)	− 0.046 − 0.881	0.087 0.114	0.600 **< 0.001**	
Year	(2018)	0.116	0.084	0.168	
Sampling period: year	(Middle: 2018) (last: 2018)	− 0.483 − 0.917	0.129 0.190	**< 0.001** **< 0.001**	
**FDis**					0.28
Intercept		0.075	0.007	**< 0.001**	
Type of tree	(Veteran)	0.015	0.007	**0.038**	
Sampling period	(Middle) (Last)	0.007 − 0.02	0.009 0.009	0.417 **0.025**	
Year	(2018)	0.0129	0.009	0.152	
Sampling period: year	(Middle: 2018) (Last: 2018)	− 0.023 − 0.036	0.013 0.013	0.069 **0.005**	
**Invertebrate attack marks**					0.32
Intercept		0.326	0.224	0.145	
Type of tree	(Veteran)	0.537	0.198	**0.007**	
Color	(Brown) (Green)	− 0.012 − 0.716	0.239 0.251	0.961 **0.004**	
Position	(Low)	0.493	0.204	**0.015**	
Sampling period	(Middle) (Last)	0.269 − 0.481	0.232 0.267	0.247 **0.041**	

Species richness and functional diversity were based on 40 paired trees (young and veteran) during three sampling periods in summer 2017 and 2018 (n = 238 from 40 trees). The number of invertebrate attack marks left on the artificial caterpillars placed around the trees were identified for the same periods in summer 2018 (n = 345). Bold text indicates significant relationships (P < 0.05).

**Figure 2:**
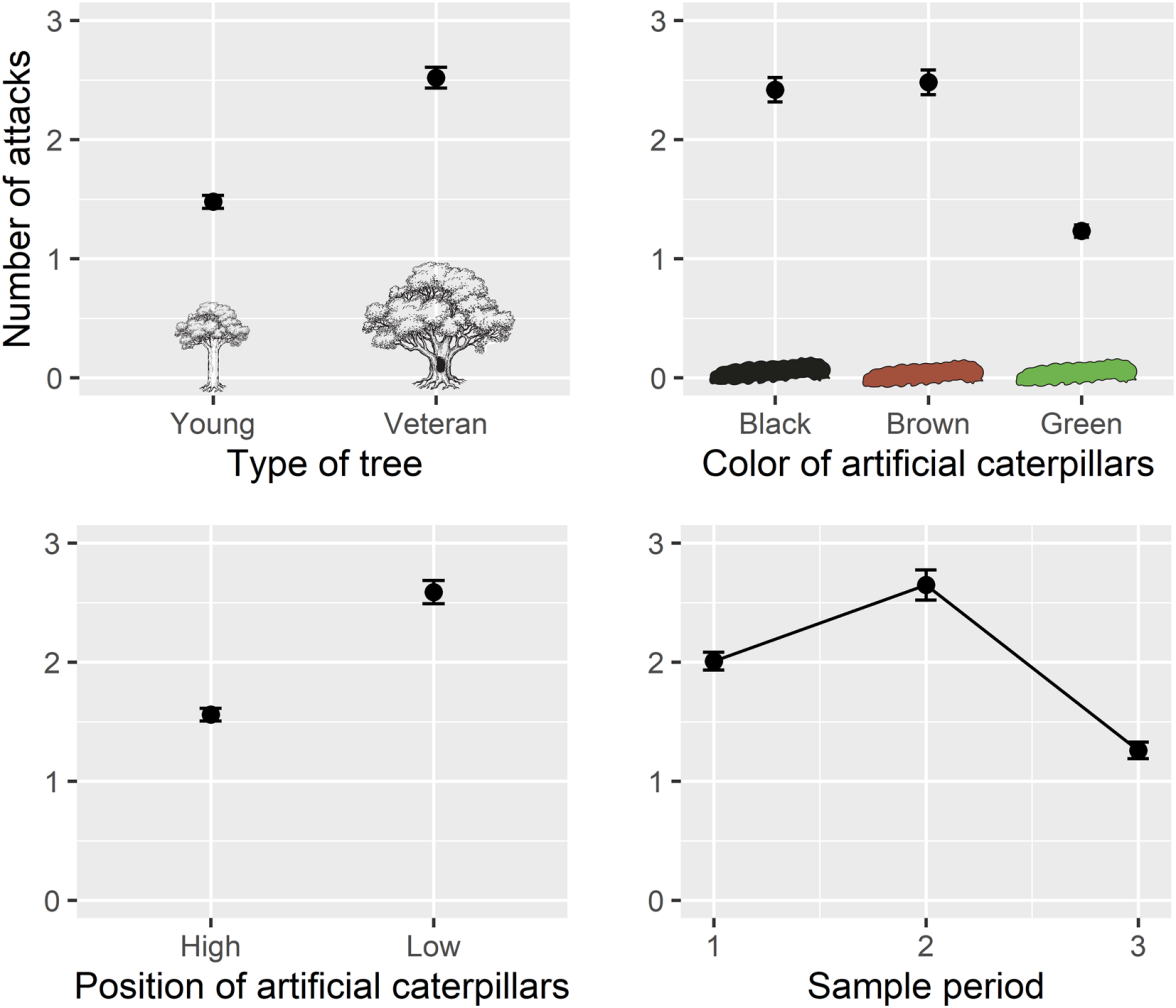
Estimated invertebrate attack rates on the artificial caterpillars from the Generalized liner mixed effect model with Negative binomial error distribution (± SEM; n = 345). Artificial caterpillars were subjected to a 30-day exposer, being collected and replaced once a month from May to August 2018, at the same time as the window trap samples were collected. A summary of the model is presented the Table 1 (Ill. by Matthew Cooper).

In total we placed out 720 artificial caterpillars, however we found that many (52%) of them were either missing or damaged to the point of obscuring other attack marks. Despite this, the remaining caterpillars were well balanced within the experimental setup. We found that arthropods attacked 47% of the remaining artificial caterpillars (n = 345). The optimal model that predicted the number of arthropod attack marks on the artificial caterpillars included the type of tree, the color of the caterpillars, the position of caterpillars and a seasonal effect (Table 1, Fig. 2). Attack rates were higher around veteran trees than young trees (P = 0.007). Green caterpillars were attacked less than caterpillars with other colors (P = 0.005), and all caterpillars were attacked more when they were placed at ground level (P = 0.016). There was also a decrease in attacks towards the end of the summer (P = 0.041).

## Discussion

In the present study, we found that our predictions that there would be higher predatory beetle diversity (both taxonomic and functional) and invertebrate attack rates around veteran oaks were supported. We also found that the diversity of predatory beetles and invertebrate attack rates decreased in parallel through the season, indicating that they were interrelated. Veteran trees have been identified as a hotspot for invertebrate diversity in Northern Europe^19^ and have a greater diversity of beetles associated with them than younger trees^15^. Building on these finding, our results are the first to show that both the diversity of natural enemies and invertebrate predation rates were higher around veteran trees than young trees, given an otherwise similar habitat. Our results considered in conjunction with each other, provide strong evidence to support the conclusion that veteran trees are a source of natural enemies.

Habitat heterogeneity is important for predation by natural enemies, as it increases the possibility of multiple invertebrate enemies to coexist^11,12^ and has been linked to a greater diversity of invertebrate predators^22,33^. The coexistence of natural enemies with complementary traits has been found to be the most important predictor of pest control by natural enemies^10^. Müller et al.^17^ attributes the finding that veteran trees had greater diversity of beetles associated with them than young trees to the structural heterogeneity proved by the veteran trees. As the size and age of a tree increases so does its structural heterogeneity, and this increases the number of micro-habitats available for shelter and potential prey species^34,35^. In our study it is likely that the structural heterogeneity proved by the veteran trees allowed for the coexistence of natural enemies and promoted complementary trait diversity, which lead to the higher predation rates that we observed around veteran trees. It should be noted however, that we did not determine the identity of the invertebrate predators attacking the artificial caterpillars, and it is therefore unknown to what extent our findings were a result of attacks from specialized predators that are dependent on veteran trees and more generalist predators that were attracted to veteran trees for shelter and as a source of prey. It is likely that it was the later, as it has been shown that ground beetles are important predators in Northern European forests^26,36,37^. In either case however, veteran trees provide resources that are beneficial for invertebrate predators and are lacking or of lower quality in younger trees^17^.

Although this study focused on the dichotomy between veteran and young trees at rather small spatial scales (200 m or less), other research has established that the immediate surroundings and the wider landscape around the veteran trees can influence invertebrate diversity^22,38–41^ and that large scale anthropogenic factors such as urbanization gradients influence predation rates by natural enemies^12,26^. In contrast, research within agroecosystems has found that strips of non-agriculture area with resources for natural enemies reduce pests and crop plant damage independent of landscape complexity^42^. It is clear that veteran trees should not be considered independent units because invertebrate diversity has been shown to be influenced by habitat connectivity at spatial scales up to 25 km^22,38,39^, but the interaction between local and regional landscape effects with predation around veteran trees needs more research.

The findings that green artificial caterpillars were attacked less by arthropod predators and that artificial caterpillars were attacked more when they were placed at ground level are interesting results that have implications for pest control with natural enemies and future research with artificial caterpillars. It is likely that green caterpillars were less detectable due to lower contrast with the background^36^ and that ground dwelling predators were responsible for the increase in attack marks on artificial caterpillars placed at ground level. It has been found that visual signals have a strong influence on hunting arthropods^43–45^, and artificial prey coloration had an effect on invertebrate attacks in Northern Europe^36^ but less so in the tropics^31^. This difference may be due to the dominance of ants in tropics, which are more chemically oriented and therefore less influenced by the prey coloration^31^. Certain ant species such as *Lasius brunneus* and *L. ferrugineus,* are associated with tree hollows in Europe^46^, but ants were only observed in high numbers at three of the study trees (one veteran and two young). On the other hand, predation by beetles has been observed to be influenced by prey coloration, although this has been found to vary between even related species^47^, and ground beetles are likely the dominant invertebrate predator attacking artificial caterpillars in Northern European forests^26,36,37^. The conclusion that beetles were the dominant predator attacking the artificial caterpillars in our study is further supported by the fact that we found ground beetles in our traps, and as discussed previously, invertebrate attack rates paralleled the trapping data of predatory beetles. It would, however, be beneficial to know the identity of the invertebrate predators so that these findings could be more clearly integrated into an understanding of predation by natural enemies.

Finally, it should be noted that a large portion of the artificial caterpillars placed out during this study were found to be missing or damaged to the point of obscuring other attack marks. It does not appear that this influenced the results related to invertebrate attacks because the remaining caterpillars were well balanced within the experimental setup. We presume that the missing and damaged caterpillars were a result of bird attacks^29^. Our study was not designed to measure avian predation and the relatively short distance between the two types of trees likely allowed birds to easily move between them. The influence of veteran trees on avian predation is clear avenue of future research, but a different study design would be needed to further explore this relationship.

## Conclusions

Our results that species richness and complementary trait diversity of invertebrate predators responded in parallel through the season with predation rates and were higher around the veteran trees clearly indicate that veteran trees are a source of natural enemies. Veteran trees are valuable because of their cultural significance^16^ and their importance for biodiversity^19^. They increase the structural complexity of landscapes^35^ and based on our results, their communities may contribute to invertebrate pest control. These results give clear incentive to protect veteran trees and their associated communities. Veteran trees also provide additional contributions of benefits to people that are both economic and cultural^48–50^. Hartel et al.^51^ goes on to state that wood-pastures with veteran scattered trees provide a model ecosystem for the sustainable integration of food production and biodiversity conservation. Protecting and valuing veteran trees and their communities and reintegrating them into agricultural systems is an essential step towards a more sustainable system of management and has the possibility of enhancing the wellbeing of people while promoting biodiversity.

## Material and methods

In order to test our predictions, we established a balanced experimental design where we chose 20 veteran oaks in the central distribution of oaks in Southern Norway from the Norwegian database of veteran oaks^51^ and matched them with 20 young oaks from the nearby surroundings. We used stratified random sampling to include veteran trees in forest and open landscapes (n = 12 and 8, respectively), had trunks with circumferences of 2 m or greater (measured at the height of 130 cm) and had young oaks in the immediate surroundings. Young oaks were within 200 m of the focal veteran oak, had similar immediate surroundings (e.g. openness, sun exposure and surrounding tree species) and were at least 50 m from any other veteran oak. The mean circumference of the veteran oaks was 283 cm (200–405 cm) and mean circumference of the young oaks was 74.5 cm (25–148 cm). The trees were originally identified within 500 × 500 m blocks, which we will refer to as the sampling blocks^51^. Twelve pairs of trees were nested in clusters of three within a sampling block, but all tree pairs were more than 100 m apart. All trees were within a 30 km radius of the city of Larvik.

**Figure 3:**
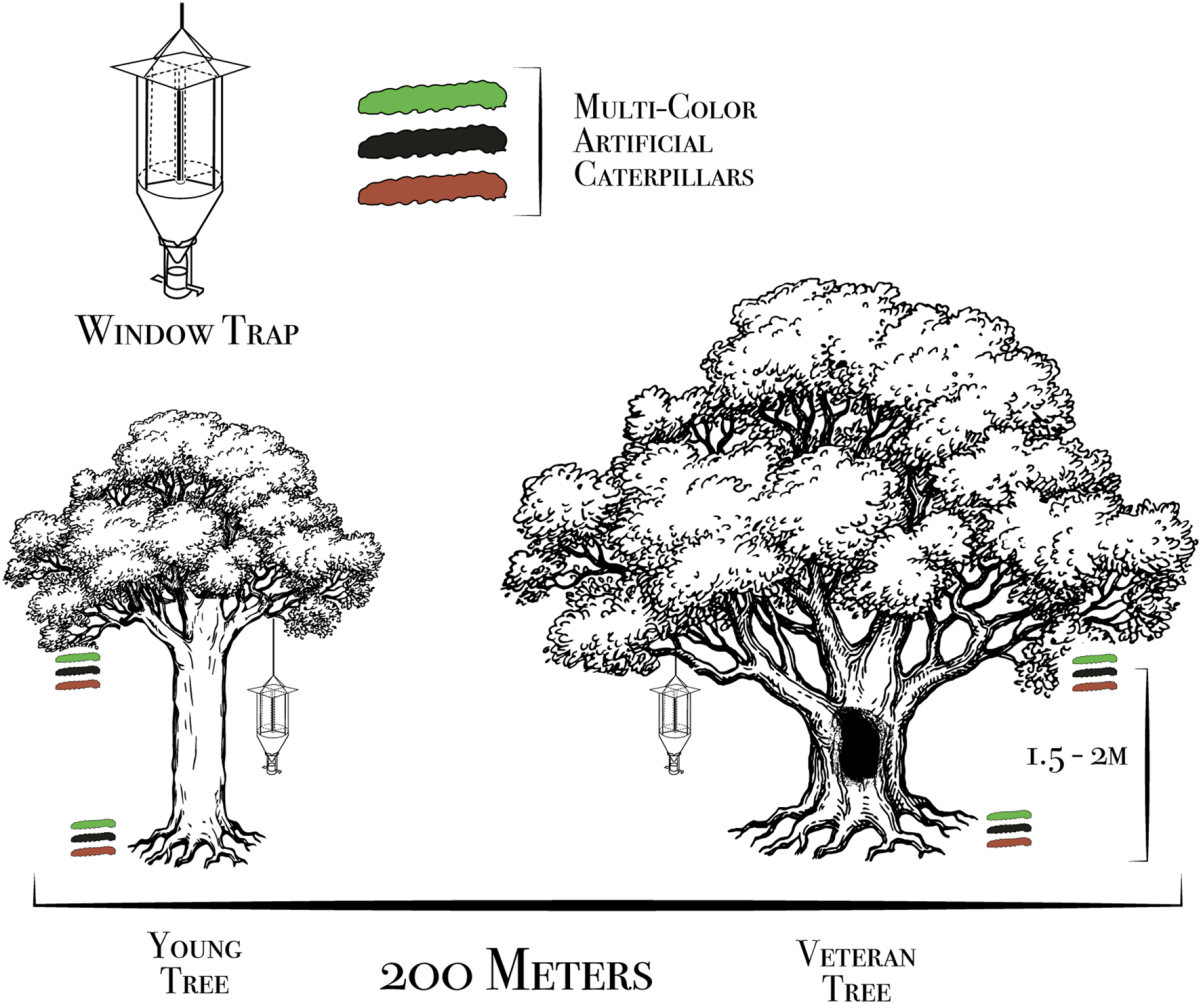
A figure of the study design that we used to measure the contribution of veteran oak invertebrate comminutes to predation by natural enemies. We measured predatory beetle diversity with window traps and predation rates with attack marks left on artificial caterpillars around 20 pairs of young and veteran oaks in Southern Norway. The window traps were active through the summer in 2017 and 2018 and artificial caterpillars were placed out in the summer 2018. The artificial caterpillars were secured to a natural attachment sites 2–4 m from the trunk of the focal tree (Ill. by Matthew Cooper).

To measure the functional and taxonomic diversity of predatory beetles, we sampled the beetle communities around the focal tree with flight intercept traps over the course of summer 2017 and 2018. The traps were made of two intersecting 20 × 40 cm windows with a funnel below leading to a vial containing propylene glycol, water (4:1 mixture) and a drop of detergent used as a surfactant. The traps were hung from a branch in the canopy of the focal tree and were placed out in May and emptied once a month until August (Fig. 3).

Predation rates were measured with artificial caterpillars made from plasticine (JOVIE) formed into 20–30 mm long and 5 mm thick cylinders by hand. A metal wire (Ø 1.1 mm) extending from the core of each caterpillar were used for attachment. Six artificial caterpillars were placed 2–4 m from the trunk of the focal tree and split equally by two positions: 1.5–2 m and at ground level (0–10 cm). The caterpillars were attached to a natural site of attachment (branch or stem). Although it has been shown that the plant species had little effect on invertebrate predation of artificial caterpillars^31^, the caterpillars was either attached to a branch of the focal tree or to a branch of a nearby deciduous tree. Three colors (green, brown and black) were chosen to mimic the variety of lepidoptera larvae commonly found in Scandinavian forests (Fig. 3). Artificial caterpillars were subjected to a 30-day exposer, being collected and replaced once a month from May to August 2018, at the same time as the window trap samples were collected. The location of the caterpillars remained the same through the experiment, but to avoid bias the colors were randomized within the location so that the order was not same between sampling periods.

Attack marks on the artificial caterpillars were documented in the field and verified in the lab. They were identified as being made by either arthropods, birds, small mammals or an unknown source based on a key provided by Low et al.^29^, and counted for each taxonomic group. In total we placed out 720 artificial caterpillars, but 375 were found to be either missing or unidentifiable, presumably due to bird attacks^29^. Despite this, the remaining caterpillars were well balanced within the experimental setup. There were 162 caterpillars remaining around the veteran trees and 183 around the young trees, 187 reaming at the high and 158 at the low location, and regarding the different colors there were 110 black, 116 brown and 119 green caterpillars. However, the number of caterpillars remaining through the sampling period did decrease as the summer progressed: we collected 177 caterpillars in the first period, 96 in the second period and 72 in the last period.

All beetles collected in the flight intercept traps were identified to the species level following the taxonomy of The Norwegian Biodiversity Information Centre^52^ by an expert. Following the protocol set by Wetherbee et al.^24^ species were classified as predators based on both adult and larvae diets, and adult trait information (body length, relative eye size and peak activity date) was collected from literature or calculated from available material (Table 2). Functional diversity was subsequently calculated based on all traits. Since functional diversity indices are sensitive to missing trait information, we verified that at least 80% of all species in the functional groups had trait information^53^. All species that were excluded as a result of lack of data were rare in the data set (abundance less than 5). We chose to use functional dispersion (FDis) to measure functional diversity because it accounts for species abundances, it can be calculated for multiple traits, and species richness has limited effect on it^54^. FDis is a measure of dispersion in trait space and is calculated as the mean distance of species to the centroid of the community and is weighted by abundances^54^.

**Table 2:** Traits included in our measure of functional diversity (trait information is from adult beetles).

Trait	Link to predation	Type/unit of measurement	Collection source
Body length	Closely linked to many life history traits such as life span and dispersal ability, and it influences the amount and composition of resources used	Millimeters	Literature
Relative eye size	Linked to prey recognition as well as hunting strategy	Mean eye circumference divided by length (measured in pixels)	Photogrammetric analysis
Peak activity date	More species being active throughout the season will increase phenological overlap with prey species and decrease intraguild predation	Year days	Literature and predictions from GBIF data

Prior to statistical analysis, we followed the steps for data exploration outlined by Zuur et al.^55^. Statistical analysis was carried out in R version 3.4.0^56^. Species richness and FDis were calculated with the *dbFD* function in the ‘FD’ package^54,56^. FDis was calculated using a Gower dissimilarity matrix and the "cailliez" correction method^54,57^. All models were created with the function *glmmTMB* from the package ‘glmmTMB’^58^. The following predictor variables were initially included in all models: whether the tree was veteran or young, the sampling period (early, mid or late), whether the tree was in an open landscape or a forest, a land use gradient, the tree cover density in a 50 and 100 m radius of the focal tree, and the circumference of the focal tree. Additionally, the sampling year was included in the beetle diversity models and the color and location of the artificial caterpillars were included in the invertebrate attack model. The best model was chosen with backward model selection based on Akaike information criterion (AIC) and non-significant predictors were removed (P > 0.05)^55^. We also determined the best error distribution and random effect structure by comparing the AIC of candidate models using the *AICtab* function in the package ‘bbmle’^59^. We compared three different random effect structures to deal with spatial correlation between the tree pairs (the veteran/young tree pair, the sampling blocks and a crossed random effect of tree pair and sampling block) and a model with no random effect, and found that a random intercept model with sampling block as the random effect was the best random effect structure for all models. The coefficient of determination (pseudo R^2^) was calculated for the models using the r.squaredGLMM function in the MuMIn package^60^.

We modeled species richness of predatory beetles with a Generalized liner mixed effect model with Poisson error distribution. We found that arthropod attack marks were overdispersed, and used a Generalized liner mixed effect model with Negative binomial error distribution with the “NB2” parameterization (variance = µ(1 + µ/*k*) to deal with the additional dispersion^58,61^. Although FDis is bound between 0 and 1, in our dataset it was approximately normally distributed, so we used a Linear mixed model with Gaussian error distribution to model it. The data also appeared to have slight zero inflation, but the Linear mixed effect model had a lower AIC and was chosen as our final model^62^. The final models were checked for patterns in the residuals, influential observations, and spatial and temporal structure that was not accounted for by the model^55^. The following packages were also used for data manipulation, statistical analysis and graphical visualization: ‘lattice’^63^, ‘ggplot2’^64^, ‘dplyr’^65^.

## Data Availability

The datasets generated during and/or analyzed during the current study are available from the corresponding author on reasonable request.
